# Systematic Identification and Assessment of Therapeutic Targets for Breast Cancer Based on Genome-Wide RNA Interference Transcriptomes

**DOI:** 10.3390/genes8030086

**Published:** 2017-02-24

**Authors:** Yang Liu, Xiaoyao Yin, Jing Zhong, Naiyang Guan, Zhigang Luo, Lishan Min, Xing Yao, Xiaochen Bo, Licheng Dai, Hui Bai

**Affiliations:** 1 Research Center for Clinical & Translational Medicine, Beijing 302 Hospital, Beijing 100039, China; liuyang@bmi.ac.cn; 2 Science and technology on Parallel and Distributed Processing Laboratory, National University of Defense Technology, Changsha 410073, China; yinxy1992@sina.com (X.Y.); ny_guan@nudt.edu.cn (N.G.); zgluo@nudt.edu.cn (Z.L.); 3 Huzhou Key Laboratory of Molecular Medicine, Huzhou Central Hospital, Huzhou 313000, China; zhongjing1003@126.com (J.Z.); malisha362@126.com (L.M.); yaoy333@126.com (X.Y.); 4 Beijing Institute of Radiation Medicine, Beijing 100850, China; boxc@bmi.ac.cn; 5 No. 451 Hospital of PLA, Xi’an 710054, China

**Keywords:** breast cancer, library of integrated network-based cellular signatures, gene set enrichment analysis, drug target, DNA methylation, Cancer Gene Census

## Abstract

With accumulating public omics data, great efforts have been made to characterize the genetic heterogeneity of breast cancer. However, identifying novel targets and selecting the best from the sizeable lists of candidate targets is still a key challenge for targeted therapy, largely owing to the lack of economical, efficient and systematic discovery and assessment to prioritize potential therapeutic targets. Here, we describe an approach that combines the computational evaluation and objective, multifaceted assessment to systematically identify and prioritize targets for biological validation and therapeutic exploration. We first establish the reference gene expression profiles from breast cancer cell line MCF7 upon genome-wide RNA interference (RNAi) of a total of 3689 genes, and the breast cancer query signatures using RNA-seq data generated from tissue samples of clinical breast cancer patients in the Cancer Genome Atlas (TCGA). Based on gene set enrichment analysis, we identified a set of 510 genes that when knocked down could significantly reverse the transcriptome of breast cancer state. We then perform multifaceted assessment to analyze the gene set to prioritize potential targets for gene therapy. We also propose drug repurposing opportunities and identify potentially druggable proteins that have been poorly explored with regard to the discovery of small-molecule modulators. Finally, we obtained a small list of candidate therapeutic targets for four major breast cancer subtypes, i.e., luminal A, luminal B, HER2+ and triple negative breast cancer. This RNAi transcriptome-based approach can be a helpful paradigm for relevant researches to identify and prioritize candidate targets for experimental validation.

## 1. Introduction

As a heterogeneous disease, breast cancer is the most frequently diagnosed cancer in women and the second leading cause of cancer death among females worldwide, accounting for 25% of all cancer cases and 15% of all cancer deaths [1,2]. Traditionally, breast cancer prognosis and classification have relied on analysis of tumor morphology and expression of three markers, i.e., estrogen receptor (ER), progesterone receptor (PR) and human epidermal growth factor receptor2 (HER2). These proteins also serve as targets for specific treatment [3]. Triple-negative breast cancers (TNBC), which refers to an absence of the expression of ER, PR and HER2, accounts for approximately 15%–20% of all diagnosed breast cancer cases. TNBC is more likely to affect younger women, African-Americans, Hispanics, and/or those with a BRCA1 gene mutation [4,5,6]. In addition, TNBC is more aggressive than other types of breast cancer and is associated with poorer survival than non-TNBC, owing to more frequent relapse in TNBC patients with residual disease [7,8].

Despite major advances in ER-positive or HER2-amplified breast cancers, which can be targeted by drugs such as tamoxifen and trastuzumab (Herceptin), there is no targeted therapy currently available for TNBC. Cytotoxic chemotherapy [4] is the mainstay of treatment since pathological complete responses after chemotherapy are more likely in TNBC than in non-TNBC [7,8]. However, research shows only 31% of TNBC patients experience pathological complete responses after chemotherapy [9], emphasizing the urgent need to explore potential gene targets for development of effective therapeutics and gene therapies.

Large-scale genomics initiatives such as The Cancer Genome Atlas (TCGA), and other complementary omics data are providing a growing list of genes that are causally involved or playing biologically and pathologically compelling roles in breast cancer, especially in TNBC [10,11,12,13,14,15]. Well studied therapeutic targets for TNBC include those proteins in proliferative and survival-dependent pathways, such as epidermal growth factor receptor (EGFR) [16,17], vascular endothelial growth factor receptor (VEGFR) [18], JAK2/STAT3 [19] and PI3K [20,21,22,23], and those control the cell cycle and the DNA damage responses such as Poly-ADP Ribose Polymerase (PARPs) [24,25]. However, the initial results of candidate compounds targeting these proteins from clinical trials [3,26,27,28,29,30] remain modest, highlighting the importance of systematic target discovering, assessing and prioritizing for biological validation and therapeutic exploitation.

RNA interference (RNAi) is an endogenous process that regulates expression of genes and corresponding proteins to maintain homeostasis in diverse organisms. In addition, RNAi therapy is emerging rapidly for personalized cancer treatment [31,32]. RNAi has also been invaluable research tool for unraveling critical genes and pathways involved in cancer development, growth and metastasis and has identified critical tumor-type specific gene targets for chemotherapy [33]. Importantly, RNAi can be combined with transcriptional profiling and used in applications such as identifying molecular biomarkers and discovering new potential targets for drug treatment [34]. However, existing studies mainly focus on knocking down a small set of genes from single cancer cell line of interest to observe typical phenotype changes. In addition, those using transcriptome analysis generally make inferences directly from the statistically significant dysregulated gene sets without taking the complex interactions into consideration.

Fortunately, the emerging of the Library of Integrated Network-Based Cellular Signatures (LINCS) data brought us with new opportunities to evaluate the cellular status from the aspect of whole genomic expression profiles after being perturbed by all kinds of perturbagens including RNAi reagents. Based on the L1000 technology [35], LINCS has recently provided open access to 1,328,098 gene expression profiles generated from 77 different cellular contexts including human primary and cancer cell lines, upon genetic knockdown of a total of 4372 genes. Ever since, the data landscape has changed substantially, making it possible for the first time to address comprehensive, objective and in depth target discovery and assessment. Therefore, there is a clear need for a systematic, objective, data-driven gene target discovery and further assessment of such gene lists, based on information integrated from different disciplines and sources, with the goal of prioritizing genes for further detailed biological validation studies.

**Figure 1:**
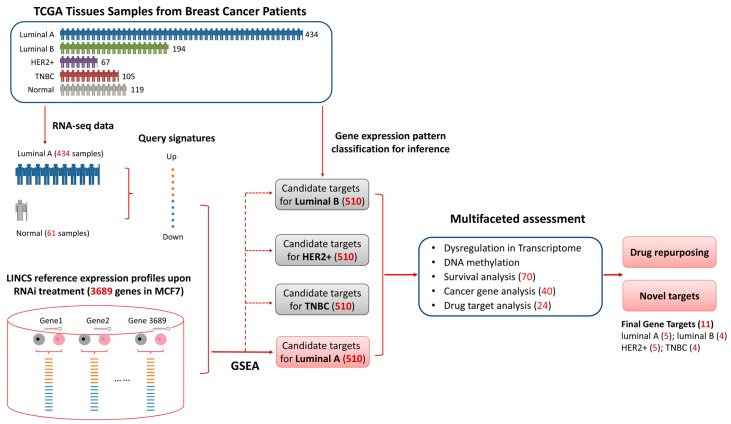
Workflow of candidate therapeutic target identification and multifaceted assessment. Luminal A phenotype specific signatures were calculated using TCGA gene expression data of luminal A breast cancer and corresponding normal samples. The signatures were then queried against Library of Integrated Network-Based Cellular Signatures (LINCS) MCF7 RNAi gene expression profile of 3689 genes using gene set enrichment analysis. Genes negatively connected to the phenotype were considered as candidate targets for luminal A. Then the targets were inferred to other three subtypes (i.e., luminal B, HER2+, TNBC) based on gene expression pattern analysis and further validated with transcriptome analysis, methylation analysis, cancer gene analysis and drug target analysis. Detailed information is provided in Materials and Methods.

Here, we demonstrate such a systematic, unbiased and objective computational approach that combines the RNAi transcriptome based target identification and multifaceted assessment. We applied our approach to identify and assess the candidate gene targets for different breast cancer subtypes, i.e., luminal A, luminal B, HER2+ and TNBC. The workflow with the computational identification, annotation scheme and assessment criteria is summarized in Figure 1 (Further information on the data sets and analysis is provided in Materials and Methods). We first establish the reference gene expression profiles from breast cancer cell line MCF7 upon genome-wide RNAi of a total of 3689 genes, and identified breast cancer query signatures using RNA-seq data generated from tissue samples of clinical breast cancer patients in the Cancer Genome Atlas (TCGA). Based on gene set enrichment analysis, we identify genes that when knocked down could significantly reverse the transcriptome of breast cancer state. By carrying out unbiased multifaceted assessment, including gene expression pattern characterization, survival analysis, DNA methylation, Cancer Gene Census, and chemoinformatics, we have been able to extrapolate and prioritize the most therapeutically promising targets within the gene list for further experimental work. In addition, we suggest potential alternative repurposing indications for known drugs and identify potentially novel proteins that have been poorly explored with regard to the discovery of small-molecule modulators.

## 2. Materials and Methods

### 2.1. Data Source

#### 2.1.1. Gene Expression and Methylation Data

mRNA expression data and methylation data for breast cancer patients were downloaded from UCSC Cancer Genomics Browser [36]. The dataset (including TCGA_BRCA_exp_HiSeqV2-2015-02-24 for mRNA expression data and TCGA_BRCA_hMethyl27-2015-02-24, TCGA_BRCA_hMethyl450-2015-02-24 for methylation data) contains 1241 samples composed of 142 basal like, 434 luminal A, 194 luminal B, 67 HER2+, 119 normal tissue and 285 undefined under the taxonomy of PAM50 [37] for breast cancers. Of all the TCGA data, 120 samples have negative expression level of ER, PR and HER2. Samples in basal like and undefined group were selected, resulting in 105 samples as TNBC (Supplementary materials Table S1). A total of 919 samples were kept for final analysis, including 434 luminal A, 194 luminal B, 67 HER2+, 105 TNBC and 119 normal tissues.

#### 2.1.2. Clinical Survival Data

Survival data for the breast cancer patients were downloaded from UCSC Cancer Genomics Browser [36]. Overall survival (OS) (in days) and vital status (LIVING or DECEASED) were provided in the dataset.

#### 2.1.3. LINCS Dataset

Publicly available LINCS L1000 level three data (q2norm) were downloaded from lincscloud (http://www.lincscloud.org) with permission. Gene knockdown expression data generated from breast cancer cell line MCF7 were used for correspondence. This dataset contains 53,763 gene expression profiles involving 12,792 RNAi reagents targeting 3689 genes and 2922 untreated profiles on 165 plates as control (Supplementary Materials Table S2). The L1000 technology originally measures expressions of 978 landmark genes to infer the entire transcriptome via linear regression. Each profile in LINCS contains 22,268 probe sets, which roughly correspond to the Affymetrix Human Genome U133A Array (HG-U133A).

#### 2.1.4. Cancer Gene Census

Cancer Gene Census [38] dataset aims to identify the mutated genes that are causally implicated in oncogenesis. The latest released dataset (accessed on Sept. 30th, 2016), which contains 602 cancer related genes roughly accounting for more than 1% of all human genes, were downloaded.

#### 2.1.5. Drug Target

Drug target information was obtained from DrugBank 5.0 [39]. The latest released DrugBank database (accessed on Sept. 1st, 2016) contains 8206 drug entries including 1991 FDA-approved small molecule drugs, 207 FDA-approved biotech (protein/peptide) drugs, 93 nutraceuticals and over 6000 experimental drugs. In total, 4253 protein targets were included in our analysis.

### 2.2. Candiate Target Idenfication Pipeline

Connectivity map uses Gene Set Enrichment Analysis (GSEA) [40] to connect a signature with reference database. To generate phenotype specific gene signatures for luminal A, hierarchical clustering was first performed to exclude outlier using 1000 most-variable genes as determined by variation. Then differential analysis was conducted with R/Bioconductor package limma [41]. Phenotypic gene signature was generated by selecting top up- and down-regulated 250 genes.

To generate the reference database, the LINCS RNAi data were processed by merging the replicates of the same shRNA reagents and calculating the log fold change via subtracting the control untreated gene expression profiles. shRNA reagent with best knocking-down effect (lowest gene expression of corresponding gene) was selected to represent the silencing effect of this gene. The probe sets in LINCS data were converted to corresponding genes using annotations from HG-U133A platform and the overlapped genes were kept for GSEA of the two datasets.

The luminal A specific signature was queried against every LINCS RNAi perturbation gene expression profile using weighted-GSEA to calculate an enrichment score estimating the correlation of gene knock-down and the phenotype. The enrichment score was normalized to a connectivity score ranging from −1 to 1. Genes with connectivity score less than −0.7 were considered as candidate gene targets. These targets are used to infer for the other three breast cancer subtypes and further validated by transcriptome analysis, methylation analysis, cancer gene analysis and drug target analysis.

### 2.3. Differential Expression Analayis

Differential gene expression analysis for the four breast cancer subtypes was conducted with R/Bioconductor package limma [41]. Note corresponding normal sample of a specific tumor sample has sampleID with the same prefix and ending with “11” according to TCGA barcode definition (e.g., TCGA-A7-A0D9-11 is corresponding normal sample of TCGA-A7-A0D9-01). Before differential gene expression calculation, outliers were removed based on hierarchical clustering result of cancer and corresponding normal samples for each subtype. Analysis was limited to the 12,555 overlapping genes between TCGA RNA-seq data and LINCS L1000 data.

### 2.4. Methylation Analysis

TCGA methylation data for breast cancer includes two different arrays, the Illumina HumanMethylation27 BeadChip (27k array) and the Illumina HumanMethylation450 BeadChip (450k array). The 450k array contains 485,577 probes covering 99% of RefSeq genes, while the 27k array only contains 27,578 probes. The 450k array is an extension of 27k array and contains most probes of the latter array. Thus, only the 450k array was used for analysis. The value range −0.5 to 0.5 provided in hgHeatmap methylation data was converted it to beta value by offsetting 0.5. Beta value was further converted to M value with beta2m function in R package wateRmelon. M value was used for differential methylation analysis with R/Bioconductor package limma. Probes with False Discovery Rate (FDR) value less than 0.05 and an average beta value difference of at least 0.2 between cancer and normal group was considered as differentially methylated CpG sites. The probes were then converted to genes with annotations contained in the methylation dataset.

### 2.5. Survival Analysis

Breast cancer samples were classified into “high expression” and “low expression” group using median of the gene expression level as the group′s cut point. Survival analysis was performed using Kaplan-Meier method [42] with R package survival. Significance of the two groups were determined with the log-rank test. Hazard ration (HR) was calculated using Cox proportional-hazards regression model implemented in coxph function of R package survival. To reduce false negative, tertile and quartile cut-off strategies which dived the samples into three and four groups respectively based on gene expression were also used. Survival analysis was only performed for the group with “highest” and “lowest” gene expression. All differences were considered statistically significant at the level of *p* < 0.05.

### 2.6. Gene Functional Annotation

Gene Ontology (GO) and Kyoto Encyclopedia of Genes and Genomes (KEGG) pathway enrichment analysis were performed with DAVID functional annotation tool [43]. Gene functional classification was performed with PANTHER [44]. Protein product of each gene was assigned to a single functional class.

## 3. Results

### 3.1. Characteristic Pattern of Gene Expression among Breast Cancer Subtypes

Traditionally breast cancer classification relies on the expression of three markers, i.e., ER, PR and HER2. Gene-expression profiling has been used to dissect the complexity of breast cancer and to stratify tumors into intrinsic gene-expression subtypes, associated with distinct biology, patient outcome, and genomic alterations [45]. Characterization of gene expression patterns facilitates the identification of specific signature that distinguishes each subtype.

TCGA now contains multi-omics data for 33 different tumor types, with an average of several hundred patient samples for each cancer type. Breast cancer samples are well characterized in TCGA, including genomic, transcriptomic, proteomic data for 1098 cases at the time of our analysis. Therefore, we first collected RNA-seq derived transcriptome data of breast tissue samples of 119 normal and 800 tumors from TCGA patients and analyzed the gene expression patterns with regard to the four major breast cancer subtypes, i.e., luminal A, luminal B, HER2+ and TNBC.

**Figure 2:**
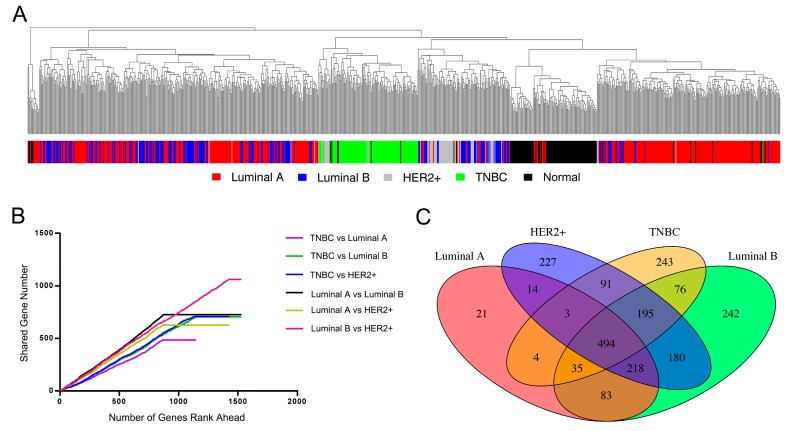
(**A**) Hierarchical clustering dendrogram of 919 breast samples (434 luminal A, 194 luminal B, 67 HER2+, 105 TNBC and 119 normal tissue) using 1000 most-variable genes as determined by variation; (**B**) Overlapping number of top ranking differentially expressed genes between any two cancer subtypes; (**C**) Venn diagram of differentially expressed genes for four subtypes.

Hierarchical clustering was used to display the expression patterns of 1000 most-variable genes [46] in the 919 breast tissue samples. Individual dendrogram branches are colored according to the strongest correlation of the corresponding tumor with the subtype centroid already defined in TCGA. As shown in Figure 2A, samples that clustered together were generally according to its pathological classification with a few exceptions. It is obvious that luminal A samples were divided into two groups, with one half of tumors clustering near each other on the right branch of the dendrogram and the other half clustering with a majority of luminal B samples on the left branch. It is noteworthy that almost all TNBC subtype samples (green branches) that showed the strongest correlations with each other are all contained within the middle branch of the dendrogram in individual tight cluster. The HER2+ and luminal B distinction was less clear, though a certain portion of HER2+ tumor samples (grey branches) within the middle branch of the dendrogram showed the strongest correlations with each other. We also performed hierarchical clustering for all samples using the 500 luminal A signature (Supplementary Materials Figure S1), and the result was consistent with above observation.

After removing outliers (Supplementary Materials Figure S2–S5) for four breast cancer subtypes, we next identified the differential expression genes of each subtype using limma, calculating *p* value, logFC (fold change), moderated *t*-statistic, False Discovery Rate (FDR) value for each gene (Supplementary Materials Table S3). For simplicity, we considered genes with absolute logFC greater than 2 and FDR less than 0.05 as differentially expressed genes. As a result, the number of differentially expressed genes is 872 for luminal A, 1523 for luminal B, 1422 for HER2+ and 1141 for TNBC. We ranked the genes based on the absolute value of moderated t-statistic (larger moderated t-statistic means more differentially expressed) and calculated the shared differentially expressed genes between any two subtypes when the number of top ranking gene increases (Figure 2B). In general, any two subtypes shared approximately at least half of the differentially expressed genes (slope larger than 0.5). It was obvious that luminal A and luminal B subtype have more shared differentially expressed genes (black line with the largest slope), indicating their transcriptome similarity. Luminal B and HER2+ subtype shared the most differentially expressed genes (red line) in total, partly because their number of differentially expressed genes are the largest under our criteria. Notably, TNBC is distinctive from the other three subtypes since the curves for TNBC with other subtypes have the shallowest slopes (purple, green and blue line). These results were consistent with the gene expression patterns characterized by hierarchical clustering.

We then calculated the number of shared differentially expressed genes among four subtypes. The four subtypes shared a total of 494 differentially expressed genes, while the number of subtype-specific differentially expressed genes is 21 for luminal A, 242 for luminal B, 227 for HER2+ and 243 for TNBC (Figure 2C). This was consistent with the above analysis results and further confirmed the transcriptome similarity of luminal A with other subtypes, as well as the distinctive transcriptome of TNBC. We performed functional annotations for the TNBC-specific differentially expressed genes with DAVID. Top enriched GO BP (Biological Process) terms are cell-cell signaling, synaptic transmission, transmission of nerve impulse, neuron differentiation, etc. Top enriched KEGG pathways are glycine, serine and threonine metabolism and drug metabolism (Supplementary Materials Table S4). Collectively, the gene expression pattern analysis and further statistics characterized the extent of transcriptome similarity of four breast cancer subtypes, which provides foundation for extrapolation of predicted gene targets using genome-wide RNAi transcriptome from a single luminal A subtype.

### 3.2. Identifying Candidate Gene Targets for Breast Cancer Luminal A Subtype

To predict candidate targets, we followed the typical connectivity map paradigm using GSEA [40] methodology, which generally composes three steps, i.e., a reference database composed of gene-expression profiles derived from the treatment of cultured human cells with a large number of perturbagens; a list of genes as query signature, which can be obtained by differential expression analysis between disease and normal state; a pattern-matching algorithm that scores each reference profile for the direction and strength of enrichment with the query signature. Theoretically, a negative score means the genes when knocked down may reverse the cancer transcriptome to normal state, which can be used as candidate gene targets for RNAi therapy.

At the time of our analysis (Sept. 30th, 2016), LINCS L1000 contained 2922 control versus 53,763 gene expression profiles upon genome-wide RNAi treatment in a single breast cancer cell line MCF7, which were used as reference database. Pathologically, MCF7 is grouped into luminal subtype A [47]. With consideration of cell line correspondence and evaluation accuracy, we used the RNA-seq data of 434 luminal A breast cancer tissue samples from TCGA versus 61 normal samples to generate the luminal A specific signature. To compose a signature, 250 most significantly up and down-regulated genes were selected based on moderate t-statistic ranking in prior differential expression analysis (Supplementary Materials Table S5). Functional annotations of these genes found that the 500-gene luminal A specific signature was enriched in cell cycle related GO BP terms such as nuclear division (GO:0000280), mitosis (GO:0007067), M phase (GO:0000279), cell cycle phase (GO:0022403) and KEGG pathways such as cell cycle (hsa04110) (Supplementary Materials Table S4). Up-regulated genes, such as COL10A1, MMP11, NEK2, PAFAH1B3, KIF4A are highly related to breast cancer [48,49,50,51,52].

**Figure 3:**
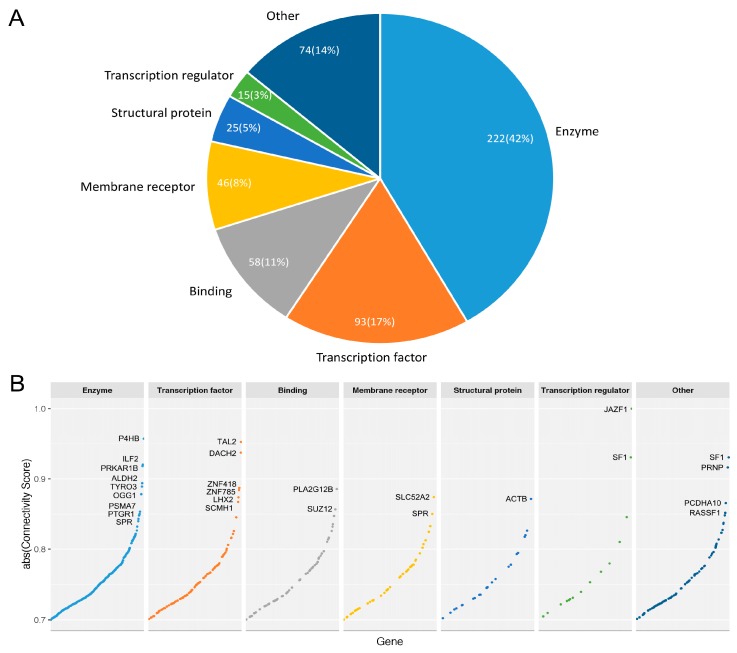
(**A**) Functional classes of protein products from the 510 candidate genes. Enzymes (predominantly kinases) and transcription factors constitute over half the candidate genes. A total of 14% of proteins, here labelled “Other”, fall into classes including transporters, cytokines, splicing factors, kinase activator, etc.; (**B**) Absolute connectivity score of candidate genes in each functional group. Genes with absolute connectivity score above 0.85 are labeled with gene symbols.

This signature was then used to query against the LINCS MCF7 expression profiles of 3689 genes upon RNAi treatment respectively. The pattern matching procedure resulted in a number of weighted enrichment score (ESs) ranging from −0.625 to 0.479. ES was then normalized to a connectivity score ranging from −1 to 1 [40]. Therefrom, we obtained a list of 510 genes with a connectivity score less than −0.7 as preliminary candidate targets for breast cancer luminal A subtype (Supplementary Materials Table S5). The 510 candidate genes were converted to HGNC standard gene symbols first. After removal of duplicates (SLC38A1 and SAT1), we classified the 510 genes into major functional classes with PANTHER [44] (Figure 3A, Detailed information in Supplementary Materials Table S6). In PANTHER, the 510 genes were mapped to 533 proteins. For simplicity, we assigned every gene product to a single functional class. The main functional classes represented in the candidate genes are as follows: enzyme (42%), transcription factor (17%), binding (11%), membrane receptor (8%), structural protein (5%) and transcription regulator (3%) (Figure 3A). Of all the enzymes, 56 are protein kinases, accounting for 10% of all candidate gene products. We also compared the connectivity score of genes with regard to their functional groups. Obviously, the enzyme and transcription factor group have more genes with high connectivity score (absolute value > 0.8), indicating higher potential to restore the dysregulated transcriptome at cancer state (Figure 3B).

### 3.3. Multifaceted Assessment for Priotizing Theraperutic Targets for Four Breast Cancer Subtypes

We hypothesized the candidate targets predicted for luminal A can be extrapolated to other breast cancer subtypes by integrating multiple filters. We then performed multifaceted assessment to analyze the biological and pathological importance of 510 candidate genes for each breast cancer subtype, from the perspectives of dysregulation in transcriptome, influence of transcriptional dysregulation on patient survival, influence of genomic abnormal methylation on gene expression, critical roles in other cancer pathogenesis, and also as novel drug targets or for repositioning purposes. In this way, we further prioritized the 510 candidate genes to screen out the targets with most potential for experimental validation.

#### 3.3.1. Dysregulation in Transcriptome and Determinant Roles in Survival

We first evaluate the expression levels of 510 genes in transcriptomes of breast tumor samples from each breast cancer subtype. Genes with logFC above 1 and FDR value less than 0.05 were considered significantly up-regulated. We illustrate the distribution of 510 genes according to their expression levels in transcriptomes and the ratio of distribution in each subtype (Supplementary Materials Figure S6). Notably, in the transcriptomes of tumor samples of luminal A, luminal B, HER2+ and TNBC, there were only 37, 50, 71, and 58 significantly up-regulated, and 59, 94, 85, and 74 significantly down-regulated candidate genes, respectively (Supplementary Materials Table S7). Consistently, the majority of candidate genes (69% to 81%) fall into the intermedia expression range regardless of the cancer subtype.

Since our evaluation was based on genome-wide RNAi transcriptomes, it is of great importance to unveil the impact of cancer transcriptomes on clinical prognosis. Overall survival (OS) and recurrence free survival (RFS) measured in days were included in TCGA for 800 breast cancer samples. Thus, we performed survival analysis to further explore the relationship of gene expression level of 510 candidate genes with survival time of breast cancer patients. Since gene expression is continuous, we applied three cut-off strategies to categorize the breast tumor samples into “high” and “low” expression group and used Kaplan-Meier method to analyze the censored data respectively. Genes with *p* value less than 0.05 and hazard ration (HR) larger than one were kept, resulting 44 genes using median cut-off strategy, 28 genes using tertile cut-off strategy and 52 genes using quartile cut-off strategy (Supplementary Materials Table S8).

Therefrom, we identified a total of 70 genes whose dysregulation in transcriptome are strongly associated with poor prognosis, especially low overall survival for breast cancer patients of all subtypes. Detailed information is provided in Supplementary Materials Table S8, and example Kaplan-Meier curves of gene MCU1 and TIAM1 are provided in Supplementary Materials Figure S7 to represent the distinctive “high” and “low” group. As shown in Supplementary Materials Figure S6 (red dots), we marked the expression level of these 70 genes for each breast cancer subtype (Supplementary Materials Table S8) and further evaluated the enrichment of these genes with high transcriptome reverse potential, i.e., absolute connectivity score above 0.8. There were only 15 poor-survival related genes with high absolute connectivity score (out of the 79 high scoring genes, hypergeometric test, *p* = 0.10). It is noteworthy that about half of the 70 poor-survival related genes were down-regulated in transcriptome (logFC < 0), which is contrary to the assumptions that patient with higher gene expression has poor prognosis. Thus, we only focused on those genes in each breast cancer subtype whose up-regulation in transcriptome are highly associated with short survival time. As a result, we found a total of 46 such genes, including 39 for luminal A, 29 for luminal B, 38 for HER2+ and 27 for TNBC, respectively. Among them, TRAPPC3, DHRS7, HYAL2, SRRT, PQBP1 and ADORA2A were of high absolute connectivity scores, and notably the latter three were valid for all subtypes (Supplementary Materials Figure S6).

#### 3.3.2. Pathogenic Importance in Cancer and Methylation at Genomic Level

Cancer Gene Census list, which is a manually curated set of genes that have mutations or other genomic abnormalities associated with cancer and are likely to be causative, as identified from genetic studies [38]. This 602-gene data set exemplifies large gene lists that have been generated from initial experimental validation. Therefrom, we found 40 out of the 510 candidate genes playing roles in specific cancers, and among them 5 genes, i.e., ALDH2, BTG1, TNFRSF14, MUC1 and STAT6, whose high expression in cancer transcriptome are associated with poor survival. Using annotations from Cancer Gene Census, 34 genes were related to breast cancer, and 4 genes, i.e., PBRM1, TP53, AKT1, CDKN1B, were in our candidate gene list.

DNA methylation of tumor suppressor genes has been the focus of numerous studies that have aimed to identify DNA methylation biomarkers of cancer [53]. Meanwhile, it is becoming clear that hypomethylation is equally important a driving force in breast cancer metastasis [54,55]. Thus, we further investigated DNA methylation status of the 23,423 genes in tumor samples of each breast cancer subtype, with the aim to identify genes that undergo significantly differential methylation at cancer state. Using TCGA methylation data generated from Illumina HumanMethylation450 BeadChip platform, we performed differential methylation analysis for 288 luminal A, 127 luminal B, 31 HER2+, and 59 TNBC tumor samples as well as 87 normal breast tissue samples. We observed a bimodal distribution of the calculated beta values, with two peaks around 0.1 and 0.9 and a relatively flat valley around 0.2–0.8 (Supplementary Materials Figure S8). In differential methylation analysis, Beta-value has a more intuitive biological interpretation, but the M-value is more statistically valid for the differential analysis of methylation levels [56]. As recommended, we used M-value to calculate differential methylation positions (DMPs) with limma. The number of normal samples was 33 for luminal A, 18 for luminal B, 6 for HER2+ and 5 for TNBC, respectively. Probes with FDR value less than 0.05 and an average Beta-value difference of 0.2 between cancer and normal samples were considered as differentially methylated probes (Supplementary Materials Table S9).

As a result, the number of significantly methylated probes (genes) was 18,747 (6909) for luminal A, 32,558 (9314) for luminal B, 20,494 (7197) for HER2+ and 6469 (3172) for TNBC. After annotation, we identified from the 510 preliminary candidate genes a set of hypermethylated and hypomethylated genes for each subtype respectively, i.e., 104 and 70 for luminal A, 128 and 111 for luminal B, 96 and 73 for HER2+, 30 and 52 for TNBC. We then focused on the hypomethylated genes that were significantly up-regulated at cancer state and calculated the enrichment of these genes with high transcriptome reverse potential (i.e., absolute connectivity score above 0.8) and/or associated with poor survival. The number of hypomethylated genes that were significantly up-regulated at cancer state was 35 for luminal A, 48 for luminal B, 41 for HER2+, 34 for TNBC. Out of the 79 genes with high transcriptome reverse potential, the number of genes that satisfy the two conditions was 4 for luminal A (TAL2, OPN3, IL20, ARID3A, *p* = 0.82), 5 for luminal B (TAL2, UMODL1, OPN3, IL20, ARID3A, *p* = 0.90), 5 for HER2+ (TAL2, OPN3, IL20, CPT1A, ARID3A, hypergeometric test, *p* = 0.79) and 4 for TNBC (UMODL1, OPN3, LDHB, ARID3A, hypergeometric test, *p* = 0.80). Out of the 70 genes associated with poor survival, the number of genes that satisfy the two conditions was 5 for luminal A (MUC1, HLA-DRA, WNT7B, XBP1, EFCAB2, hypergeometric test, *p* = 0.54), 4 for luminal B (ATG16L2, MUC1, WNT7B, XBP1, *p* = 0.92), 5 for HER2+ (C1QTNF6, NDUFS6, MUC1, HLA-DRA, XBP1, hypergeometric test, *p* = 0.69) and 4 for TNBC (CHERP, MUC1, TIAM1, GABRP, hypergeometric test, *p* = 0.71).

#### 3.3.3. Druggable Candidate Genes

To further evaluate the potential of candidate genes as novel targets as well as drug repositioning opportunities, we have identified proteins from the 510 candidate genes that are themselves targets of FDA-approved drugs or experimental compounds. These proteins, examples of drugs and associated therapeutic indications are detailed in Supplementary Materials Table S10. We have found that a total of 80 candidate proteins are targets of FDA-approved drugs and 47 are targets of experimentally validated active compounds (nutraceuticals excluded). When limited to targets of the 147 approved anti-neoplastic drugs (ATC code L01) in DrugBank (version 5.0), a total of 24 gene products currently have small-molecule drugs indicated for cancer therapy. We further calculated the druggable proteins from those of the 70 candidate genes that affect survival of breast cancer patients, and found 17 were targets of known drugs. For example, ICAM1 is targeted by natalizumab, which is approved for treatment of multiple sclerosis. ADORA2A is targeted by multiple drugs such as oxtriphylline for treatment of the symptoms of asthma, bronchitis, COPD, and emphysema. Among these genes, only proteins of two candidate gene FPGS and POLE2 currently have small-molecule drugs indicated for cancer therapy, i.e., raltitrexed for malignant neoplasm of colon and rectum and cladribine for lymphoproliferative diseases such as hairy cell leukemia, Non-Hodgkin's lymphoma, etc. Together, these results indicate great repositioning potential of candidate genes as drug targets for anti-neoplastic purposes in breast cancer.

#### 3.3.4. Prioritized Therapeutic Targets for Four Breast Cancer Subtypes

**Figure 4:**
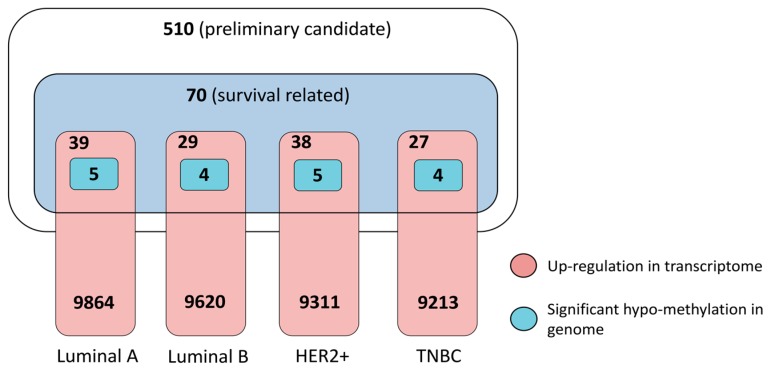
Statistical summarization of final candidate targets for four breast cancer subtypes from multifaceted assessment.

Finally, combining all of the above assessment, we obtained a small list of 11 genes, i.e., MUC1, HLA-DRA, WNT7B, XBP1, EFCAB2, ATG16L2, C1QTNF6, NDUFS6, CHERP, TIAM1 and GABRP, as the most potential targets for four breast cancer subtypes (Table 1). The number of final candidate targets for each subtype is 5 for luminal A, 4 for luminal B, 5 for HER2+ and 4 for TNBC (Figure 4, Supplementary Materials Table S11). These genes show averagely high transcriptome reverse potential (absolute connectivity score > 0.7) when knocked down by RNAi reagents, indicating feasibility as targets for developing RNAi therapeutics. Meanwhile, these genes were verified by genome and transcriptome data from tumors samples of clinical breast cancer patients as significantly hypo-methylated at genomic level and transcriptionally up-regulated, despite their distinct dysregulation levels in each subtype. Notably, we found MUC1 a commonly valid target for all four subtypes (Supplementary Materials Figure S9), XBP1 for four subtypes except for TNBC, WNT7B for the two luminal subtypes, and HLA-DRA for luminal A and HER2+. Most importantly, there are several genes that can distinguish the four breast cancer subtype as specific therapeutic targets for each subtype, including EFCAB2 for luminal A, ATG16L2 for luminal B, C1QTNF6 and NDUFS6 for HER2+, as well as CHERP, TIAM1, and GABRP for TNBC (Table 1). Among them, only GABRP has known drug targeting for the treatment of diseases such as insomnia and epilepsy, and this leaves potential for drug repurposing.

Obviously, most of the common targets are highly related to breast cancer, and their pathogenic importance in breast cancer has been experimentally validated. For example, MUC1 encodes glycoprotein Mucin 1 with extensive O-linked glycosylation of its extracellular domain and overexpression of MUC1 is often associated with colon, breast, ovarian, lung and pancreatic cancers [57]. It is a multifaceted oncoprotein which promotes growth, metastasis, and resistance to drugs in cancer [58]. Immune responses to MUC1 have been seen in breast and ovarian cancer patients and clinical studies have been initiated to evaluate the use of antibodies to MUC1 and of immunogens based on MUC1 for immunotherapy of breast cancer patients [59,60]. XBP1 functions as a transcription factor during endoplasmic reticulum (ER) stress by regulating the unfolded protein response (UPR). XBP1 is activated in TNBC and has a pivotal role in the tumorigenicity and progression of this human breast cancer subtype by controlling HIF1α pathway. In breast cancer cell line models, depletion of XBP1 inhibited tumor growth and tumor relapse [61]. WNT7B encoded Wnt7b is a Wnt ligand that has been demonstrated to play critical roles in several developmental processes. Myeloid WNT7b mediates the angiogenic switch and metastasis in breast cancer, and therapeutic suppression of WNT7B signaling might be advantageous due to targeting multiple aspects of tumor progression [62]. HLA-DRA encodes HLA class II histocompatibility antigen, DR alpha chain. It is part of the HLA class II molecule which is expressed in antigen presenting cells (APC) and plays a central role in the immune system by presenting peptides derived from extracellular proteins. This protein is generally invariable, yet research showed HLA-DRA was highly overexpressed in ovarian cancer, perhaps as a result of inflammatory events in the tumor microenvironment. The tumor cells may have compensatory mechanisms to reduce the production of functional MHC class II molecules, thus reducing immunogenicity and favoring tumor growth [63].

With regard to the specific targets predicted to TNBC, TIAM1 encodes Tiam1 (T-lymphoma invasion and metastasis 1), one of the known guanine nucleotide (GDP/GTP) exchange factors (GEFs) for Rho GTPases (e.g., Rac1) and is expressed in breast tumor cells (e.g., SP-1 cell line). Research showed ankyrin-Tiam1 interaction plays a pivotal role in regulating Rac1 signaling and cytoskeleton function required for oncogenic signaling and metastatic breast tumor cell progression [64]. GABRP encodes Gamma-aminobutyric acid receptor subunit pi, which is a component of a cell-surface receptor. Research showed GABRP stimulates basal-like breast cancer cell/ triple negative subtype migration through activation of extracellular regulated kinase 1/2 (ERK1/2). In addition, silencing GABRP in BLBC cells decreases migration, BLBC-associated cytokeratins and ERK1/2 activation [65].

## 4. Discussion

Ongoing large-scale loss of function studies like LINCS genome-wide RNAi screens combined with gene expression profiles are now producing tons of data that may offer limitless opportunities to discover the driving force underlying cancer pathogenesis. In this study, we systematically evaluated the transcriptome reverse potential of 3689 genes for breast cancer using LINCS profiling data from genome-wide RNAi perturbagens, resulting in a preliminary list of 510 candidate genes with absolute connectivity score above 0.7. Considering the inherent noise in LINCS L1000 data, we used this relative loose threshold of connectivity score to screen out the candidates, which is supposed to potentially reduce the false negatives. Altogether, this transcriptional bioinformatics method is more rational and straightforward since it evaluates the real cellular states when genes are knockdown without being confused by the uncertain inner interactions from simply inferring from the most dysregulated genes, thus hoping to provide more insights than existing methods.

In cancer research, cancer cell lines have long been used as experimental models because they generally carry the genomic, transcriptomic and proteomic characteristics of the primary tumor from which they were derived. Tumor is classified by stage and grade in clinic, since cancer cell lines that acquire indefinite growth tend to be high stage and poorly differentiated tumors, they are not entirely capable of representing the clinical spectrum of cancers at that site [66]. Thus, a critical issue is to match cell line to corresponding disease when computationally compare the expression profiles generated from clinical tissue samples of certain disease and those from the LINCS cell line. In an idea way, our reference signatures should also include those generated from breast cancer cell lines of different breast cancer subtypes other than luminal A. However, due to the limited breast cancer cell line used in LINCS project, we performed computational evaluation based on transcriptome similarity analysis of TCGA RNA-seq data generated from clinical breast tumor samples of different subtypes, and therefrom identification of the query signature of breast cancer luminal A subtype, which is in complete congruent with the subtype classification of all the LINCS reference signatures. As the gene expression pattern of luminal A is less distinctive and heterogeneous than other subtypes, we are able to extrapolate and prioritize the candidate targets for each other subtype by further integrating multifaceted assessment.

After preliminary identification, objective prioritization to a more manageable gene list is essential, as detailed biological validation of each individual target is a challenging, long and expensive process. Our multifaceted assessment takes into account of essential factors of both pathogenic importance and druggability. We have shown that, by integrating information from different large-scale research initiatives (comprising information on dysregulation in transcriptome that affect patient survival, in other cancer types, and in DNA methylation), we are able to effectively annotate a biologically and pathogenically important gene list containing potential targets for RNAi therapies. Further, by integrating information from protein functional class as targets of approved drugs and experimental active small molecules, we have also identified targets for repurposing known drugs or active chemical compounds that can be tested for activity in breast cancer models. Of particular interest for new drug discovery, we have identified 11 proteins in the final list (as highlighted in Table 1, with specific examples discussed above) that need to be examined carefully. In addition to having biological and pathogenic importance, they lack chemical compounds to modulate them, representing potential novel biological targets for chemical exploitation. Besides RNAi therapy, these targets can be addressed with small-molecule compounds through two strategies: by investing in medicinal chemistry approaches to expand the boundaries of druggability; or via the identification of alternative druggable targets within the pathway or subnetwork in question.

## 5. Conclusions

Collectively, this global, systematic transcriptome-based evaluation and objective, multidisciplinary computational assessment presented here allows for the effective, unbiased and data-driven identification and prioritization of large, biologically compelling gene lists for the purpose of RNAi therapy or drug discovery. This approach, applied here to the LINCS breast cancer genome-wide RNAi profiles an exemplar, can be adapted to any other cell line based LINCS profiles under different types of perturbagens, e.g., over expression vectors and CRISPR/Cas9 system. Meanwhile, the following multifaceted assessment can also be customized for specific purposes using annotations of interest that emerge from large-scale omics studies, functional screening initiatives or from any therapeutic area. In this way, researchers can find more reasonable driving force behind breast cancer, and significantly reduce the time of therapeutic target identification and risk in following preclinical drug development.

## Figures and Tables

**Table 1:** Final candidate gene targets for four breast cancer subtypes.

Gene Symbol	Gene Name	Luminal A		Luminal B		HER2+		TNBC		Protein Name	No. of Targeted Drugs
		CS	logFC	CS	logFC	CS	logFC	CS	logFC		
MUC1	mucin 1, cell surface associated	−0.75	3.11	−0.75	1.97	−0.75	2.33	−0.75	0.43	Mucin-1	0
HLA-DRA	major histocompatibility complex, class II, DR alpha	−0.73	0.31	-	-	−0.73	0.8	-	-	HLA class II histocompatibility antigen, DR alpha chain	0
WNT7B	Wnt family member 7B	−0.73	2.14	−0.73	1.96	-	-	-	-	Protein Wnt-7b	0
XBP1	X-box binding protein 1	−0.72	1.58	−0.72	1.68	−0.72	0.94	-	-	X-box-binding protein 1	0
EFCAB2	EF-hand calcium binding domain 2	−0.72	0.08	-	-	-	-	-	-	EF-hand calcium-binding domain-containing protein 2	0
ATG16L2	autophagy related 16 like 2	-	-	−0.77	0.19	-	-	-	-	Autophagy-related protein 16-2	0
C1QTNF6	C1q and tumor necrosis factor related protein 6	-	-	-	-	−0.77	2.23	-	-	Complement C1q tumor necrosis factor-related protein 6	0
NDUFS6	NADH: ubiquinone oxidoreductase subunit S6	-	-	-	-	−0.77	1.34	-	-	NADH dehydrogenase [ubiquinone] iron-sulfur protein 6, mitochondrial	1
CHERP	calcium homeostasis endoplasmic reticulum protein	-	-	-	-	-	-	−0.77	0.46	Calcium homeostasis endoplasmic reticulum protein	0
TIAM1	T-cell lymphoma invasion and metastasis 1	-	-	-	-	-	-	−0.71	0.68	T-lymphoma invasion and metastasis-inducing protein 1	0
GABRP	gamma-aminobutyric acid type A receptor pi subunit	-	-	-	-	-	-	−0.7	1.5	Gamma-aminobutyric acid receptor subunit pi	41

Note: CS means connectivity score; FC means fold change; “-” means the corresponding values do not reach the thresholds that define statistical significance.

## Supplementary Materials

The following are available online at www.mdpi.com/2073-4425/8/3/86/s1. Table S1: This table gives the detailed clinical information of 434 luminal A samples for TCGA breast cancer, Table S2: This table gives the detailed information of LINCS MCF7 RNAi experiments data and control untreated data, Table S3: This table gives the differential expression analysis result of limma for luminal subtype A, Table S4: KEGG pathway analysis result of luminal A signature genes with DAVID, Table S5: This sheet contains luminal A signature for GSEA calculation, i.e., top 250 up- and down-regulated genes for luminal A subtype, Table S6: Functional classification of 510 candidate genes with PANTHER, Table S7: Transcriptome analysis of 510 candidate genes for TNBC, Table S8: Information of 70 survival related genes for TNBC, Table S9: This sheet contains methylation analysis result for TNBC (gene centric), Table S10: This sheet contains DrugBank drugs which target the 70 survival related genes predicted with our pipeline, Table S11: Final candidate targets for HER2+, Figure S1: Hierarchical clustering result of 919 breast samples (434 luminal A, 194 luminal B, 67 HER2+, 105 TNBC and 119 normal tissue) using 500 luminal A specific signature, Figure S2: Hierarchical clustering result of 434 luminal A breast samples and corresponding normal samples using 1000 most-variable genes as determined by variation, Figure S3: Hierarchical clustering result of luminal B breast samples and corresponding normal samples using 1000 most-variable genes as determined by variation, Figure S4: Hierarchical clustering result of HER2+ breast samples and corresponding normal samples using 1000 most-variable genes as determined by variation, Figure S5: Hierarchical clustering result of TNBC samples and corresponding normal samples using 1000 most-variable genes as determined by variation, Figure S6: Distribution of logFC (log2-fold change) values of 510 candidate genes in transcriptome of (A) Luminal A, (B) Luminal B, (C) HER2+, (D) TNBC breast tumor samples, Figure S7: Kaplan–Meier curve of selected genes (A) MCU1 with *p* value 0.018 and HR 2.06. (B) TIAM1 with *p* value 0.022 and HR 1.57, Figure S8: Bimodal distribution of the calculated beta values, with two peaks around 0.1 and 0.9 and a relatively flat valley around 0.2–0.8, Figure S9: Venn diagram of final candidate gene targets for four breast cancer subtypes.

## Abbreviations

The following abbreviations are used in this manuscript:

TNBC	Triple Negative Breast Cancer
LINCS	Library of Integrated Network-based Cellular Signatures
GSEA	Gene Set Enrichment Analysis
TCGA	The Cancer Genomics Atlas
