# Supplementary material for: Systematic Identification and Assessment of Therapeutic Targets for Breast Cancer Based on Genome-Wide RNA Interference Transcriptomes
Source: Genes (Basel). 2017 Feb 24;8(3):86. doi: 10.3390/genes8030086 (PMC5368690; doi:10.3390/genes8030086)
Supplement: Supplementary file 1 [file genes-08-00086-s001.zip › genes-167720-sups/genes-167720-suppl.-figure-final.docx]

Systematic Identification and Assessment of Therapeutic Targets for Breast Cancer Based on Genome-Wide RNA Interference Transcriptomes

Yang Liu ^1,†^, Xiaoyao Yin ^2,†^, Jing Zhong ^3,†^, Naiyang Guan ^2^, Zhigang Luo ^2^, Lishan Min ^3^,
Xing Yao ^3^, Xiaochen Bo ^4^, Licheng Dai ^3,^* and Hui Bai ^4,5,^*

^1^ Research Center for Clinical & Translational Medicine, Beijing 302 Hospital, Beijing 100039, China; liuyang@bmi.ac.cn

^2^ Science and technology on Parallel and Distributed Processing Laboratory, National University of Defense Technology, Changsha 410073, China; yinxy1992@sina.com (X.Y.); ny_guan@nudt.edu.cn (N.G.); zgluo@nudt.edu.cn (Z.L.)

^3^ Huzhou Key Laboratory of Molecular Medicine, Huzhou Central Hospital, Huzhou 313000, China; zhongjing1003@126.com (J.Z.); malisha362@126.com (L.M.); yaoy333@126.com (X.Y.)

^4^ Beijing Institute of Radiation Medicine, Beijing 100850, China; boxc@bmi.ac.cn

^5^ No. 451 Hospital of PLA, Xi’an 710054, China

***** Correspondence: dlc21@126.com (L.D.); huibai13@hotmail.com (H.B.); Tel.: +86-572-2555800 (L.D.);
+86-010-66932251 (H.B.)

^†^ These authors contributed equally to this work


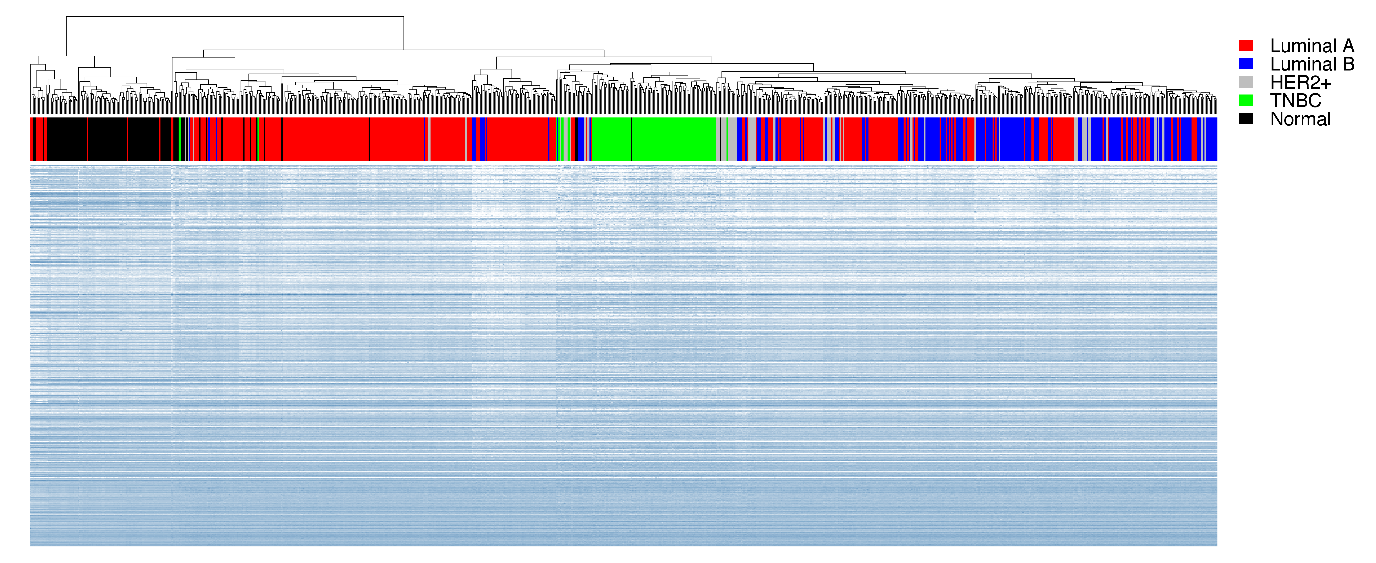


**Figure S1.** Hierarchical clustering result of 919 breast samples (434 luminal A, 194 luminal B, 67 HER2+, 105 TNBC and 119 normal tissue) using 500 luminal A specific signature.


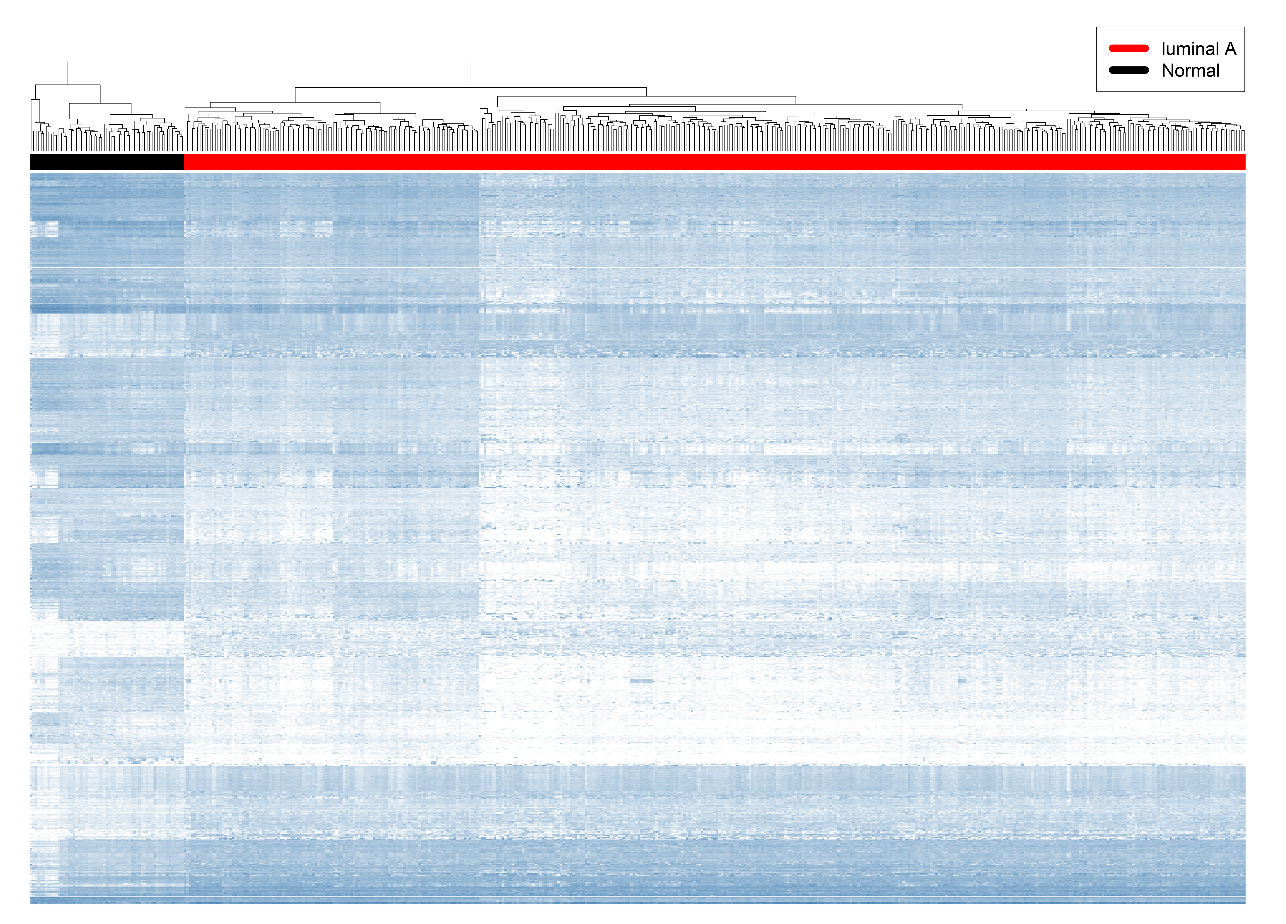


**Figure S2.** Hierarchical clustering result of 434 luminal A breast samples and corresponding normal samples using 1000 most-variable genes as determined by variation.


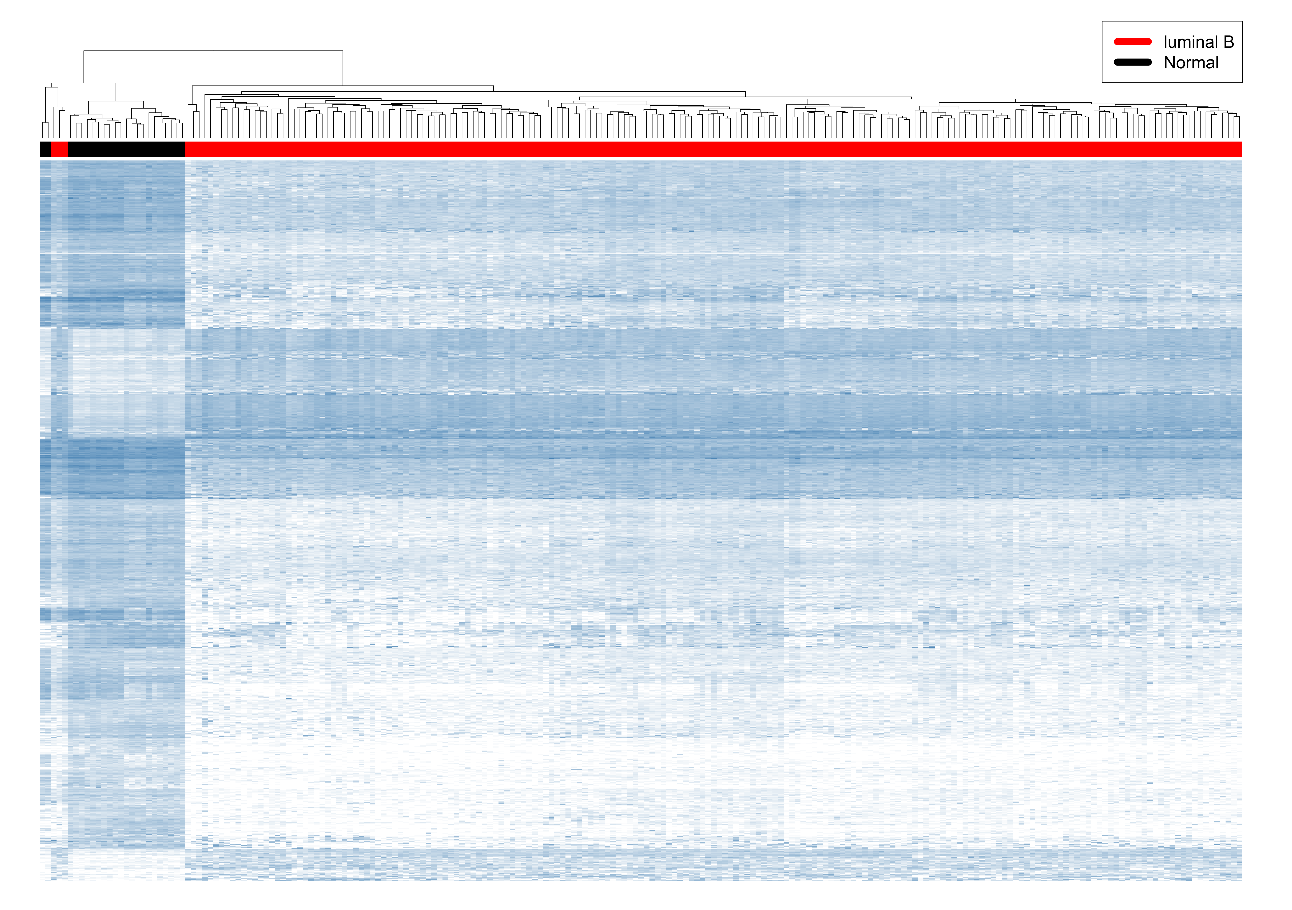


**Figure S3.** Hierarchical clustering result of luminal B breast samples and corresponding normal samples using 1000 most-variable genes as determined by variation.


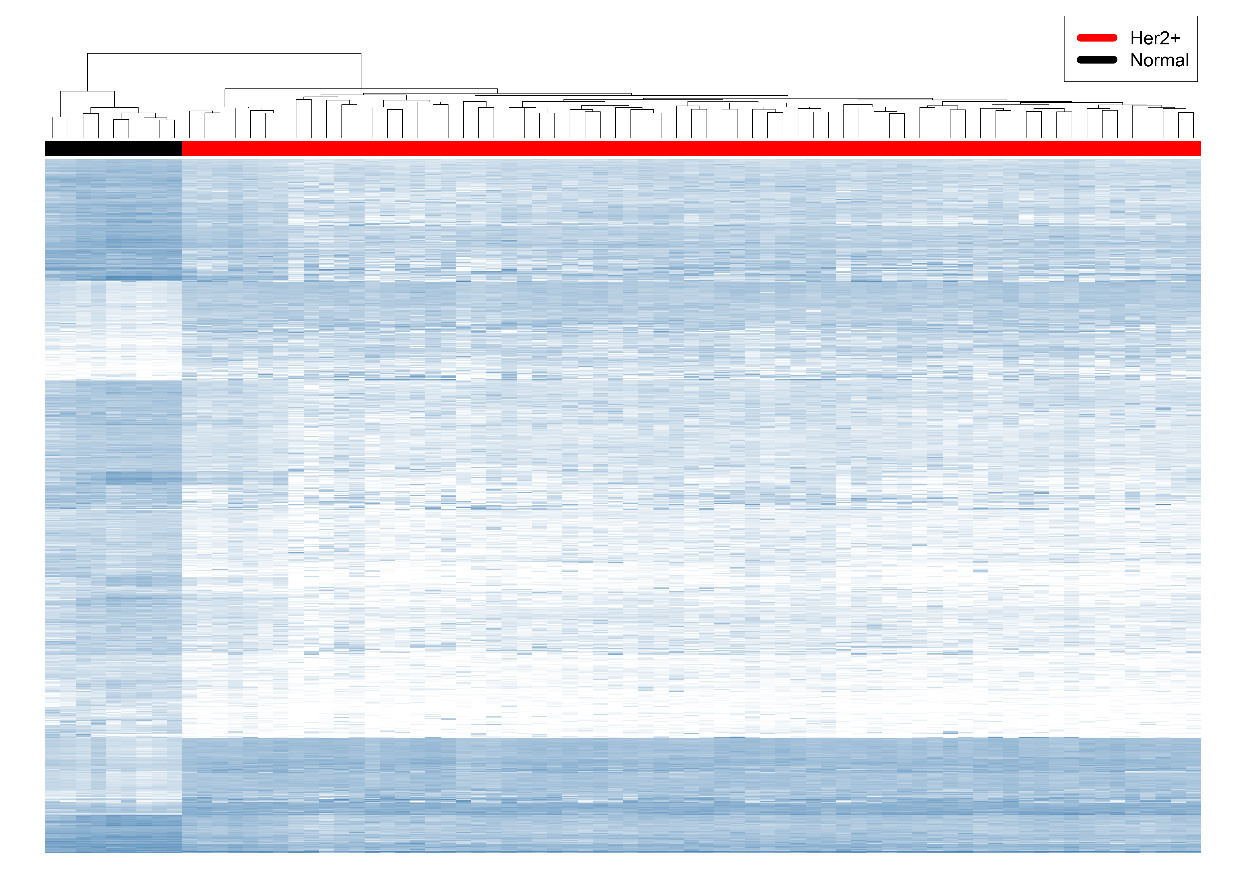


**Figure S4.** Hierarchical clustering result of HER2+ breast samples and corresponding normal samples using 1000 most-variable genes as determined by variation.


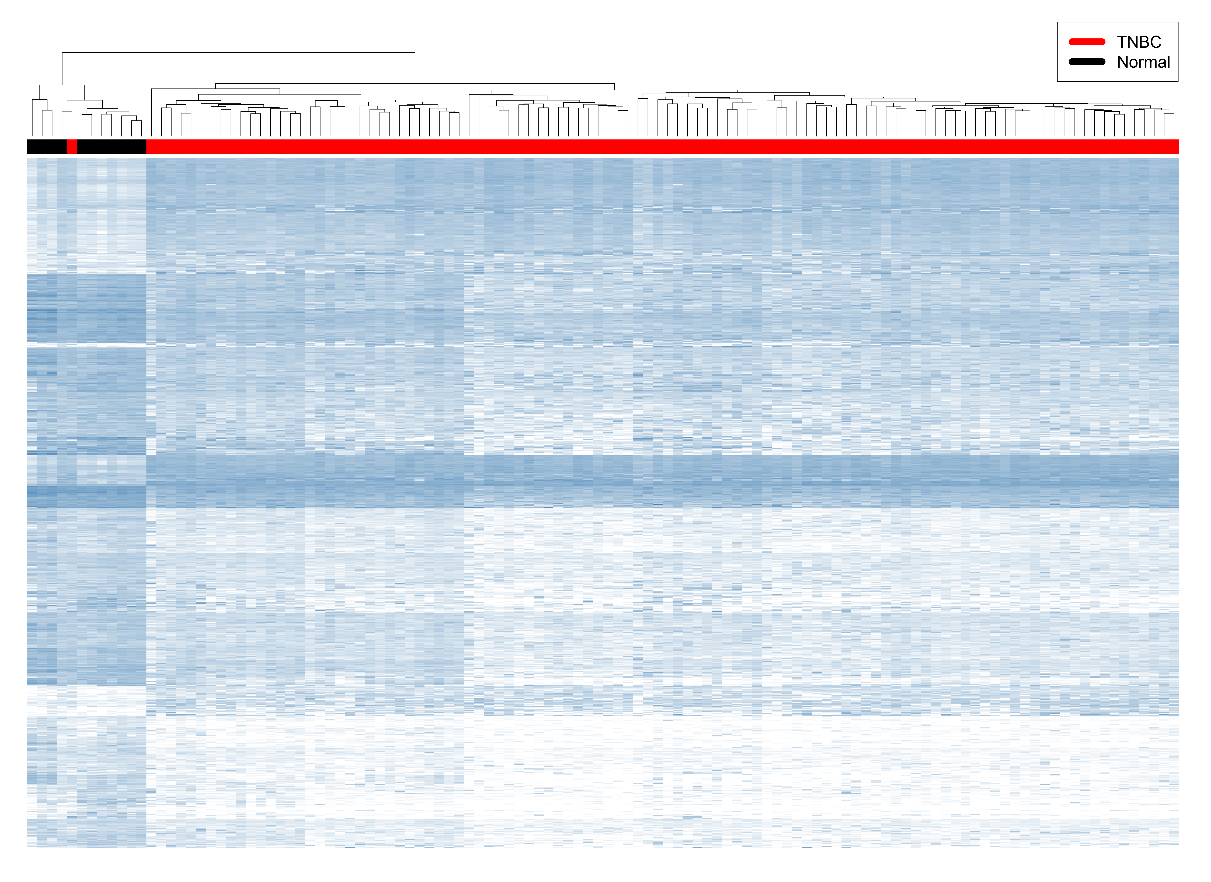


**Figure S5.** Hierarchical clustering result of TNBC samples and corresponding normal samples using 1000 most-variable genes as determined by variation.


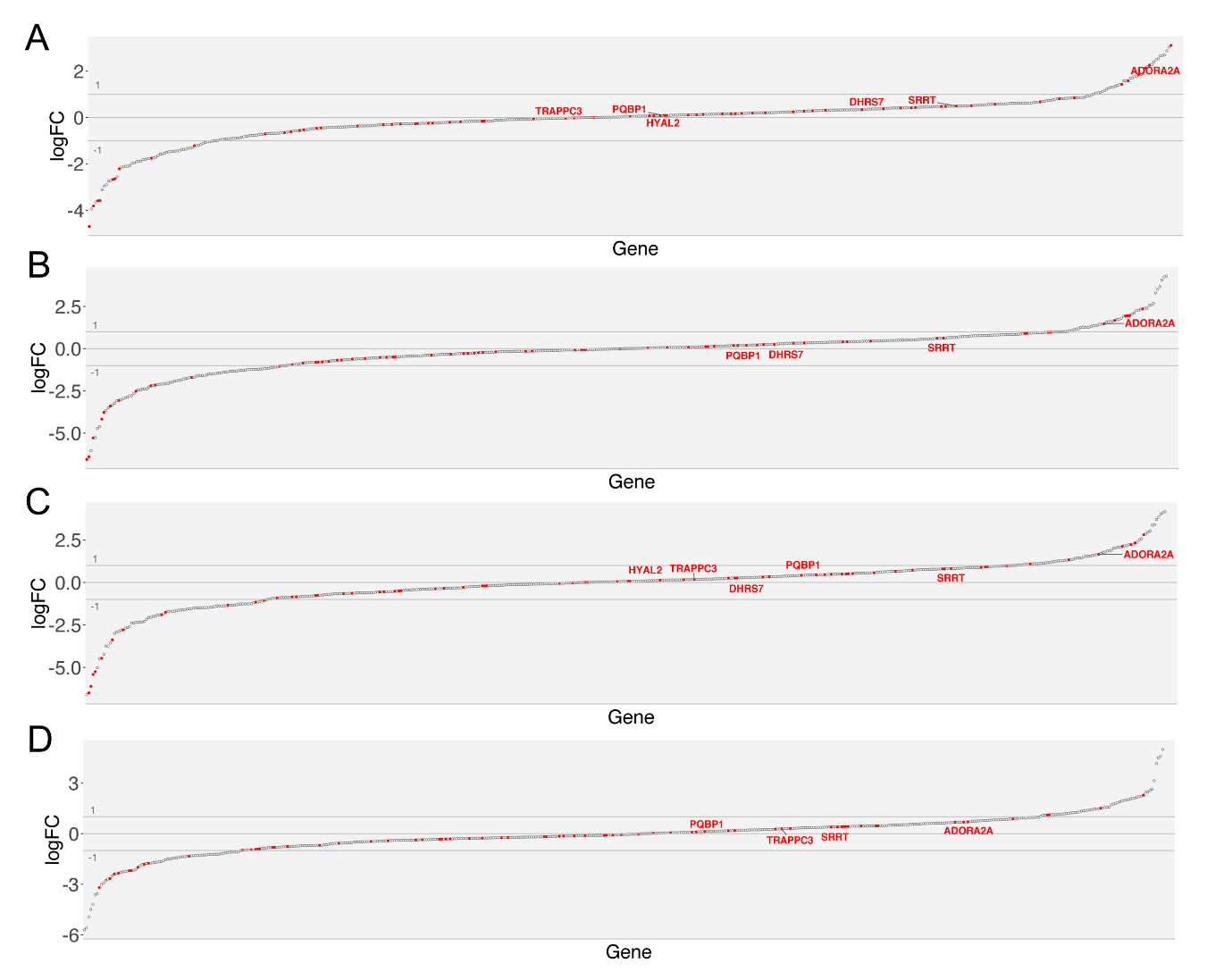


**Figure S6.** Distribution of logFC (log2-fold change) values of 510 candidate genes in transcriptome of (A) Luminal A, (B) Luminal B, (C) HER2+, (D) TNBC breast tumor samples. Genes whose dysregulation in transcriptome (high gene expression) are associated with poor survival were highlighted in red. And those up-regulated genes (logFC > 0) with high absolute connectivity score (above 0.8) were further labelled with gene symbol.


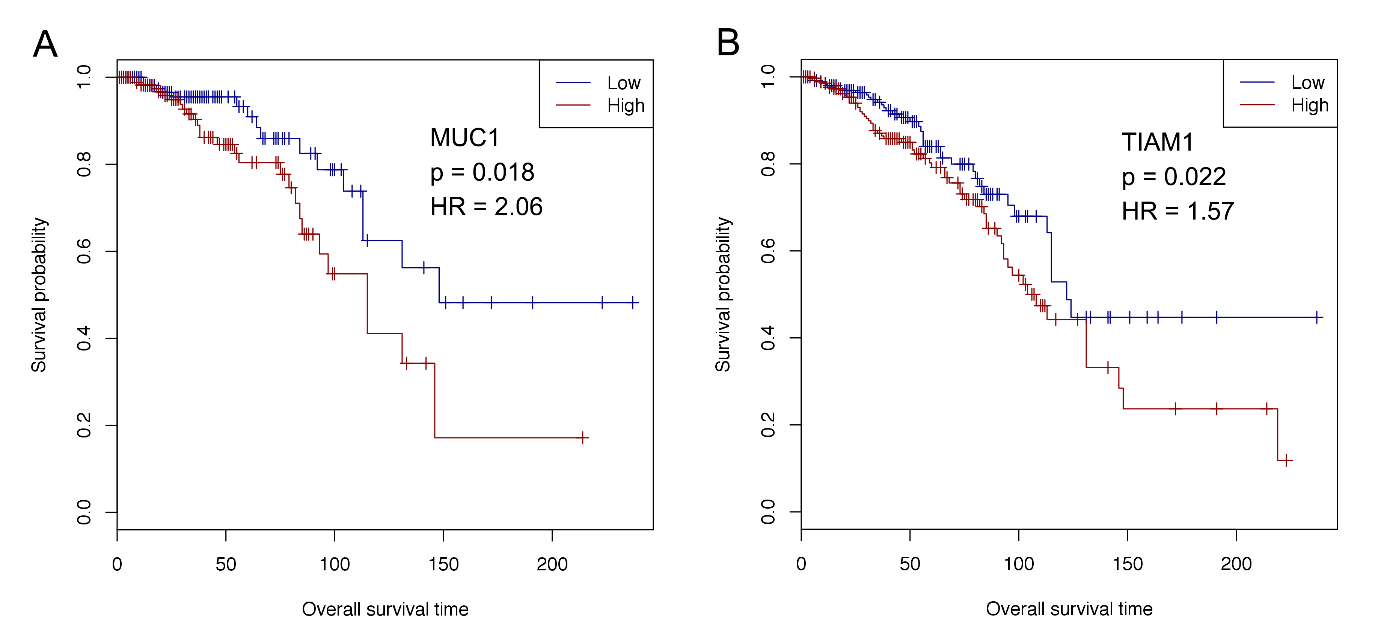


**Figure S7.** Kaplan–Meier curve of selected genes (A) MCU1 with p value 0.018 and HR 2.06. (B) TIAM1 with p value 0.022 and HR 1.57.


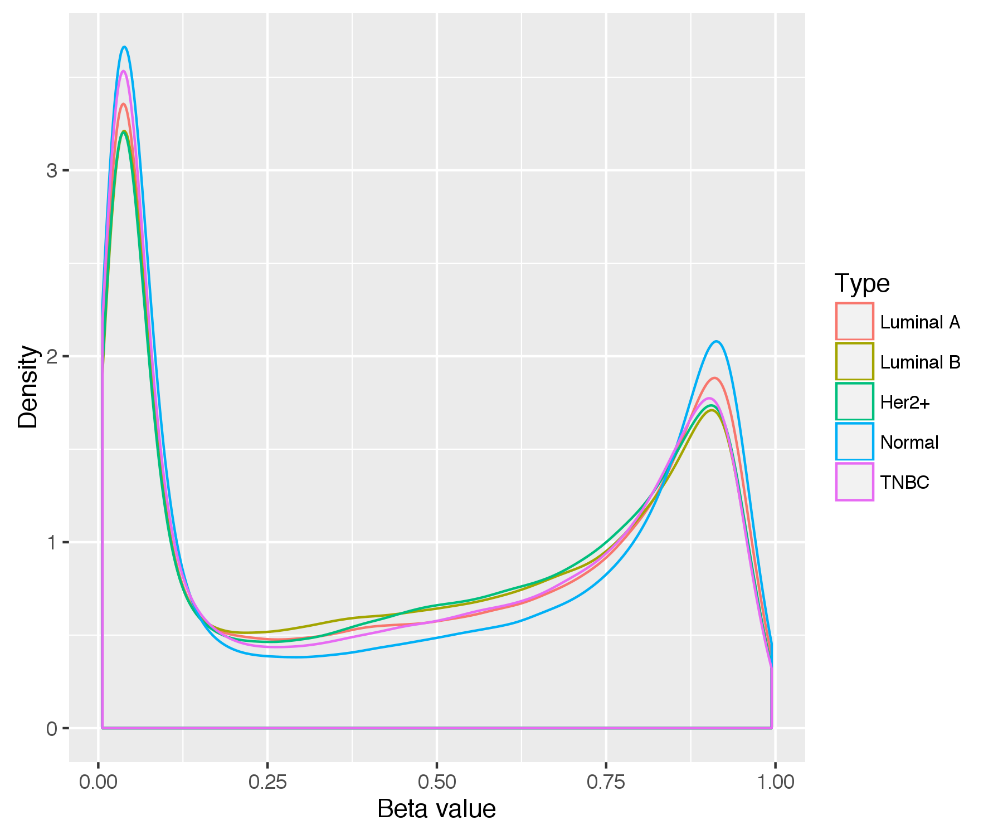


**Figure S8.** Bimodal distribution of the calculated beta values, with two peaks around 0.1 and 0.9 and a relatively flat valley around 0.2 - 0.8.


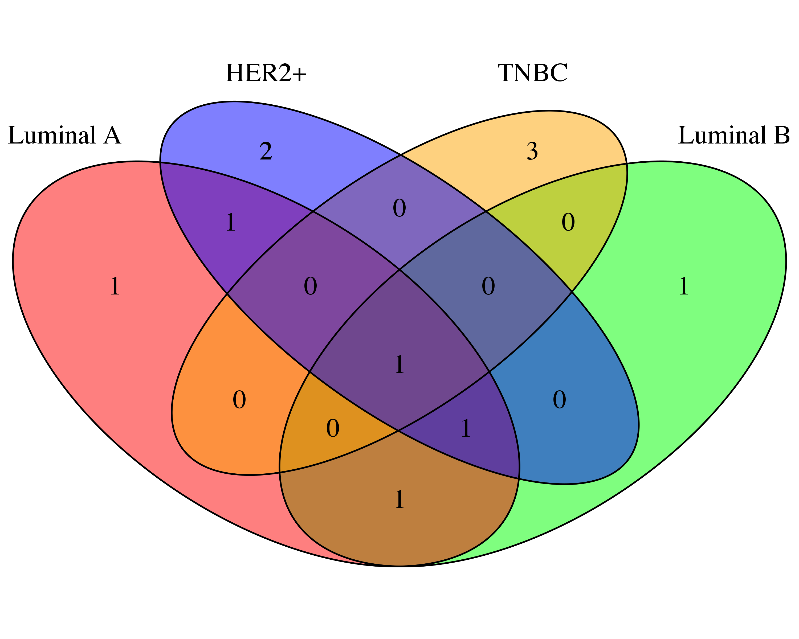


**Figure S9.** Venn diagram of final candidate gene targets for four breast cancer subtypes.
